# The epidermal growth factor receptor (EGFR) in head and neck cancer: its role and treatment implications

**DOI:** 10.1186/1748-717X-1-11

**Published:** 2006-05-02

**Authors:** Michel Zimmermann, Abderrahim Zouhair, David Azria, Mahmut Ozsahin

**Affiliations:** 1 Department of Radiation Oncology, Centre Hospitalier Universitaire Vaudois, Bugnon 46, 1011 Lausanne, Switzerland; 2 Department of Radiation Oncology, INSERM Cancer Research Institute, CRLC Val d'Aurelle, Rue Croix-Verte, 34298 Montpellier cedex 05, France

## Abstract

Epidermal growth factor receptor (EGFR) is a member of the ErbB family of receptors. Its stimulation by endogenous ligands, EGF or transforming growth factor-alpha (TGF-α) results in activation of intracellular tyrosine kinase, therefore, cell cycle progression. High levels of EGFR expression are correlated with poor prognosis and resistance to radiation therapy in a variety of cancers, mostly in squamous-cell carcinoma of the head and neck (SCCHN). Blocking the EGFR by a monoclonal antibody results in inhibition of the stimulation of the receptor, therefore, in inhibition of cell proliferation, enhanced apoptosis, and reduced angiogenesis, invasiveness and metastases. The EGFR is a prime target for new anticancer therapy in SCCHN, and other agents in development include small molecular tyrosine kinase inhibitors and antisense therapies.

## Review

Squamous-cell carcinoma of the head and neck (SCCHN) remains a challenging clinical problem, due to the persisting high rate of local and distant failure, as well as the occurrence of second primaries. Treatment for early stage disease involves usually surgery and radiation therapy (RT). Locally-advanced tumors are best treated with concurrent chemotherapy to RT, either in the definitive setting or following surgery, according to each center's expertise.

Although altered radiation fractionation and chemoradiotherapy had a favorable impact for advanced head and neck cancer patients, the outcome of patients presenting with stage III-IV SCCHN is still poor, with 5-year actuarial survival rates fluctuating between 30% and 40% in most trials [1].

Recent research efforts have attempted to exploit biologic differences that may exist between normal and malignant cells, to develop tumor-specific therapies. The epidermal growth factor (EGF) and its receptor (EGFR, ErbB-1, or HER-1) were not only shown to play an influential role in cellular growth and differentiation in healthy tissues, but also in tumorigenesis and the progression of malignant disease [2].

As well as being expressed on the surface of healthy cells, the EGFR is commonly expressed at high levels in a variety of epithelial tumors, including SCCHN. The aberrant activation of the EGFR leads to enhanced proliferation and other tumour-promoting activities, which provide a strong rationale to target this receptor.

During the past decade, intense research has initiated a new era of cancer treatment, that of molecular therapeutics. Today, the EGFR is a prime target for new anticancer therapy, with a broad range of inhibitors currently under investigation [3].

Promising preclinical studies have prompted the development of clinical trials testing EGFR inhibitors as single-agent therapy or in combination with conventional cytotoxic therapy, with response rates lower than anticipated in the advanced disease setting. The clearest benefit of EGFR-inhibitor treatment to date is noted when it is combined with RT to treat locally advanced head and neck cancer [4].

### The epidermal growth factor receptor

It was not until 1980 that Cohen et al managed to purify the EGFR [5], 15 years after the initial isolation of its ligand, EGF [6]. EGFR is a glycoprotein of 170 kDa, encoded by a gene located on chromosome 7p12 [7]. It belongs to the ErbB receptor family (EGFR or Her-1, Her-2, Her-3, and Her-4). These receptors are composed of an extra-cellular ligand-binding domain, a hydrophobic transmembrane segment, and an intracellular tyrosine kinase domain.

Binding to EGFR by its natural ligands, mainly EGF or transforming growth factor alpha (TGF-α) results in a conformational change in the receptor, which promotes homodimerization with other EGFR molecules or heterodimerization with other HER family members (especially Her-2); dimerization results in subsequent autoactivation of the tyrosine kinase from the intracellular domain of the receptor. This process will activate an intracellular signalling pathway, leading to the inhibition of apoptosis, activation of cell proliferation and angiogenesis, as well as an increase in metastatic spread potential [8].

### The radiobiological rationale

The effect of radiation on tumor-cell proliferation has been extensively studied in the setting of RT of the head and neck. Accelerated repopulation, a condition of enhanced cellular proliferation after exposure to ionising radiation, appears to be responsible, at least in part, for radioresistance of head and neck cancers. Preclinical evidence suggests that EGFR has an important role in the proliferative response to ionizing radiation, counteracting the toxic effects of RT. Mechanisms of activation may be diverse, including increased EGFR expression [9] but one key mechanism involves probably ligand-stimulated activation.

RT is able indeed to activate early the transduction signalling pathway of EGFR, through radiation-induced release of TGF-α, one of the EGFR ligands [10].

The inhibition of radiation-induced activation of the EGFR signalling pathway is one of the factors explaining the observed synergy between RT and EGFR inhibition; an increase in radiosensitivity through this pathway was demonstrated in vitro [11]. It has to be reminded that, at this point, no clear relationship has been demonstrated between EGFR expression (at least as measured by immunohistochemistry) and the level of radiation sensitization achieved with anti-EGFR. We are unable as well to identify tumors that are radioresistant by virtue of EGFR signalling, and are thus likely to become radiosensitized by EGFR inhibitors [12].

### EGFR expression in head an neck cancer

In normal cells, the expression of EGFR ranges from 40,000 to 100,000 receptors per cell [13]. In SCCHN, EGFR and its ligand, TGF-α, are overexpressed in 80–90% of cases; the corresponding magnitudes of increase are 1.7-fold (P = 0.005) and 1.9-fold (P = 0.006) respectively, when compared to controls [14]. The nature of the protein overexpression is thought to result from enhanced transcription, with no apparent change in mRNA stability; gene amplification has been observed less frequently.

TGF-α is participating in an autocrine-signalling pathway in transformed, but not in normal mucosal epithelial cells. Targeting the translation start site of TGF-α mRNA with antisense oligonucleotides decreases TGF-α protein by up to 93% and reduces cell proliferation by a mean of 76% in human cell lines [15].

EGFR overexpression is an early event in SCCHN carcinogenesis; it is already present in "healthy" mucosa (field cancerization) from cancer patients, when compared to healthy controls; this overexpression will increase steadily in parallel to observed histological abnormalities, from hyperplasia to invasive carcinoma, through dysplasia and in situ carcinoma [16].

### Prognostic value of EGFR expression

Most preclinical and clinical studies demonstrated a lower local control after radiation therapy in tumors overexpressing EGFR [17]. A former study in 140 patients with primary laryngeal squamous-cell carcinoma showed that the 5-year survival rate was 81% for patients with EGFR non-expressing tumors, compared with 25% for patients with EGFR-expressing tumors (P < 0.0001) [18]. These results were also confirmed by others [19].

A recent retrospective study [20] evaluated the EGFR expression in 155 patients with stage III-IV SCCHN accrued in the control arm of RTOG 9003 study, and received exclusive external beam RT (70 Gy in 7 weeks). A detectable expression of EGFR was found in 148/155 patients (95%), with a wide range in interindividual variability. In this study, EGFR expression was found to be independent from tumor stage or initial nodal involvement; in multivariated analysis, it showed to be an independent pronostic factor of overall survival (P = 0.006), and of disease-free survival (P = 0.003), as well as a robust pronostic factor of locoregional relapse (P = 0.002) but not of distant relapse (P = 0.50).

If quantitative evaluation of EGFR by immunohistochemistry has emerged so far as a convenient and promising marker for clinical outcome correlation, a more accurate reflection of the "activity state" of EGFR signalling status might be provided by the phosphorylated or "activated" forms of EGFR downstream signalling molecules like phosphorylated MAPK, phosphorylated AKT or Stat-3 [21,22]. They are currently actively evaluated as potential surrogate markers of EGFR signalling in clinical therapeutic trials.

### Inhibition of EGFR activity

Two complementary therapeutic strategies have been developed. The first one targets the extracellular domain of the receptor with monoclonal antibodies (cetuximab, C225, or Erbitux^®^); binding of the antibody to the EGFR prevents activation of the receptor by endogeneous ligands through competitive inhibition; it also results in internalization and degradation of the antibody-receptor complex, downregulating EGFR expression.

The second strategy targets the intracellular domain of the receptor with low-molecular-weight tyrosine kinase inhibitors (gefitinib, ZD 1839, Iressa^®^; erlotinib, OSI 774, Tarceva^®^), competing with adenosine triphosphate (ATP) for its binding site on the intracellular domain of EGFR.

These two classes of anti-EGFR agents did not meet the expectations in clinical practice when used in monotherapy, resulting more often in a cytostatic than a cytotoxic effect [23].

Combining EGFR inhibitors with conventional chemotherapy provided disappointing results so far. The Eastern Cooperative Oncology Group (ECOG) conducted a phase III study in 121 patients with relapsed or metastatic SCCHN; patients were randomized between a chemotherapy-only arm, and an arm combining cisplatin with cetuximab. Survival differences were not significant [24].

### Cetuximab and radiation therapy

In 2000, Bonner et al demonstrated in a pannel of SCCHN cell lines that the combination of cetuximab (5 μg/l) delivered simultaneously with radiation (3 Gy) resulted in a greater decrement in cellular proliferation than either treatment alone, regardless of the inherent EGFR expression of the cell line [25]. These promising results served as preclinical background for clinical investigations involving C225/radiation in human SCCHN.

The same author conducted a multinational phase III study involving 424 patients with locoregionally advanced SCCHN treated with curative intent [4]. Accrual took place between April 1999 and March 2002. Patients were randomized between definitive RT, *versus *the same RT regimen combined to weekly administration of cetuximab. Median follow-up was 54 months. Radiotherapy plus cetuximab significantly prolonged progression-free survival (hazard ratio for disease progression or death: 0.70 (0.54–0.90); P = 0.006).

The median duration of overall survival in the definitive RT arm was 29.3 months, against 49.0 months in the arm combining radiation therapy plus cetuximab (P = 0.03). The 3-year survival rate was 45% for patients receiving RT alone, and 55% for those receving RT and cetuximab.

Grade 3–5 acneiform rash was more common in the arm with cetuximab (17%) than in the RT alone arm (1%); this difference was statistically highly significant (P = < 0.001). Importantly, however, the use of cetuximab did not appear to exacerbate radiation-induced mucositis (grades 3–5: P = 0.44; all grades: P = 0.84) nor other toxicities.

This is the first study to ever demonstrate a survival benefit related to the administration of an EGFR inhibitor (cetuximab) when combined to RT in head and neck cancer, confirming the promising results provided by previous phase II clinical studies. Enthusiasm has still to be tempered, as the control arm was unfortunately RT alone and not concomitant chemoradiation.

It has to be reminded that this trial was designed in an era when radiation alone was still considered an acceptable standard in the treatment of advanced head and neck cancer patients; in the mean time, concurrent radiochemotherapy has assumed a preferred role for these patients. This trial enabled at least an unencumbered assessment regarding the capacity of cetuximab to augment radiation response and outcome without the confounding variable of chemotherapy [26].

The promising results from this phase III study will still require further cross-validation through additional trials to confirm outcome advantage for the combination of cetuximab with (chemo-) radiation therapy. A broad series of new clinical trials is currently under way [Table 1].

**Table 1:** Current phase II/III trials assessing EGFR inhibitors in combination with radiation (RT) locally advanced non-metastatic stage IV squamous-cell cancer of the head and neck

Trial	Protocol ID	EGFR inhibitor	Status	Comments
Phase III	RTOG-0522	Cetuximab	Active	Concurrent chemoRT +/- C225
Phase II	ECOG-E3303	Cetuximab	Active	Concurrent chemoRT + C225, followed C225 maintenance
Phase II	RTOG-0234	Cetuximab	Active	Surgery followed by adjuvant chemoRT (cisplatin vs. docetaxel) + C225
Phase II	NCT00140556	Erlotinib	Active	Concurrent chemoRT + erlotinib + bevacizumab
Phase II	NCT00226239	Cetuximab	Active	Induction chemotherapy with docetaxel/cisplatin + C225 followed by RT/cisplatin + C225
Phase II	NCT00193284	Gefitinib	Active	Induction chemotherapy with docetaxel/carboplatin/5-FU + gefitinib followed by RT/gefitinib +/- docetaxel

EGFR: epidermoid growth factor receptor

### Tyrosine kinase inhibitors (TKI) and RT

In preclinical studies, when gefitinib was combined to RT, strikingly greater than additive effects were observed *in vivo *[27].

At the ASCO 2005 meeting, Cohen et al. presented the results from a phase II study, integrating gefitinib into a concurrent chemoradiation regimen in patients with advanced SCCHN, followed by gefitinib alone in the adjuvant setting [28]. From the 69 patients accrued, only 42 subjects were evaluable for response, with a median follow-up of 10 months (16 patients had not yet been evaluated, the others were not evaluable for various reasons). Grade III-IV toxicities were consistent with previous chemoradiotherapy trials. Complete response rate (CR) was 88% (37/42), suggesting that this regimen might be promising for patients with advanced SCCHN.

No mature data are available regarding erlotinib in combination with (chemo)radiotherapy in advanced SCCHN.

Phase III studies will have to evaluate standard chemoradiotherapy in combination with TKIs or placebo for advanced HNSCC, as well as the potential role of these small molecules in the adjuvant setting.

## Conclusion

Despite decades of intensive clinical investigations, the outcome of patients presenting with stage III-IV HNSCC is still poor, with 5-year actuarial survival rates fluctuating between 30% and 40% in most trials. These findings underscore the need to develop novel strategies in the management of patients with advanced HNSCC.

Accelerated radiation schemes lead to an enhanced 5-year local control from 60–70%, associated with an improved disease-free survival, but with no benefit regarding overall survival [29].

Clinical trials testing combined modality therapy demonstrate that cytotoxic drugs given before (induction or neoadjuvant chemotherapy) or after (adjuvant chemotherapy) surgery or radiation do not improve significantly the local and distant control of the disease. In contrast, administering chemotherapy concurrently with radiation therapy has improved the 5-year overall survival rate by about 8%, but at the costs of increased local toxicity [30].

The magnitude of the survival benefit in favour of chemoradiation is almost identical regardless whether mono-chemotherapy or poly-chemotherapy is used. Cisplatin and 5-FU appear to be more effective than carboplatin or mitomycin C; no randomized data are available for newer cytostatic drugs like taxanes that have been shown to be effective in head and neck cancer [31].

Current chemo- and radiation therapies have reached their limits, with only minor improvements to be expected in the future; research is currently developing new treatment strategies, integrating novel targeted therapies in clinical practice.

In SCCHN, EGFR is not only an independent prognostic factor of outcome in multivariate analysis, but also a first-choice therapeutic target. The recent demonstration of a significant survival benefit when combining cetuximab with external RT is a major breakthrough in the management of SCCHN, establishing a new treatment option for locally advanced SCCHN. This trial provided also an important proof of principle that targeting a pertinent signalling pathway can enhance the radiation response of tumors. However, the improvement in the loco-regional control rate has been modest (within the range achieved with concurrent radiotherapy and chemotherapy) and more than half of patients receiving radiotherapy plus cetuximab still experienced local-regional relapse [4]. Therefore, there is a need to further improve outcome. Ongoing clinical efforts are devoted to address whether the addition of cetuximab to concurrent chemoradiation can yield a better outcome (i.e., RTOG study 0522).

At this point, it has to be reminded that cancer cells rely on several, sometimes, redundant activation pathways; EGFR is only one of them. The risk of treatment failure is real, if only one receptor is targeted, hence the interest in combining broader range tyrosine kinase inhibitors such as CI-1033, which targets all four members of the Erb family (pan ErbB).

Finally, ionizing radiation stimulates the nitric oxyde (NO) pathway as well as the production of VEGF [32,33]. Angiogenesis inhibitors bear the potential to reinforce the cytotoxic action of RT. ZD6474, a small molecule tyrosine kinase inhibitor of EGFR and VEGF, looks very promising for the future.

## Abbreviations

SCCHN: squamous-cell carcinoma of the head and neck

RT: radiation therapy

EGF: epidermal growth factor

EGFR: epidermal growth factor receptor

TGF-α: transforming growth factor alpha

RTOG: Radiation Therapy Oncology Group

ATP: adenosine triphosphate

ECOG: Eastern Cooperative Oncology Group

TKI: tyrosine kinase inhibitor

NO: nitric oxyde

VEGF: vascular endothelial growth factor

## Competing interests

The author(s) declare that they have no competing interests.

## Authors' contributions

MZ and AZ drafted the manuscript. DA helped the draft of the manuscript and participated in its design. MO conceived of the review, participated in its design, and helped the draft of the manuscript. All authors read and approved the final manuscript.
